# Engineering VIGS Vectors by Modifying Movement Proteins of the 30K Family

**DOI:** 10.1002/biot.202400584

**Published:** 2024-12-22

**Authors:** David Villar‐Álvarez, José A. Navarro, Vicente Pallas, Jesús Ángel Sanchez‐Navarro

**Affiliations:** ^1^ Instituto de Biología Molecular y Celular de Plantas (IBMCP) Universitat Politècnica de Valencia‐Consejo Superior de Investigaciones Científicas Valencia Spain

**Keywords:** 1 30K family, AMV, CMV, MP, silencing, TMV, VIGS

## Abstract

Virus‐induced gene silencing (VIGS) represents a particularly relevant tool in agricultural species for studying gene functionality. This study presents a novel approach for utilizing viruses belonging to the 30K family of movement proteins (MPs) as VIGS vectors. The method described here employs smaller inserts (54 bp or less) than those commonly used (100–500 bp). The developed strategy involves modifying 30K family MPs to introduce heterologous sequences of the gene of interest into their coding sequence. This approach enabled the successful induction of gene silencing in *Nicotiana tabacum* and *Nicotiana benthamiana*. Three representative viruses of the MP 30K family, alfalfa mosaic virus (AMV), cucumber mosaic virus (CMV), and tobacco mosaic virus (TMV) were employed. The capacity to induce gene silencing of small inserts (18–54 bp) was investigated, enabling to establish a correlation between insert size and silencing efficacy. This allowed the system to be calibrated to achieve partial silencing levels. The relationship between viral encapsidation and the level of gene silencing was also investigated, revealing that a high efficiency of viral encapsidation results in a reduction in the level of gene silencing achieved. Considering these findings, it can be concluded that the approach carried out with AMV, CMV, and TMV could be applied to other members of the MP 30K family. The MP 30K family comprises 20 viral genera and over 500 viral species, which can infect all agronomically significant plant species. Consequently, the strategy presented in this work could be applied to a wide range of relevant hosts.

## Graphical Abstract and Lay Summary

Silencing of plant genes is achieved by inserting small sequences of the target gene into the open reading frame of viral movement proteins belonging to the 30K family. Depending on the size of the insert, the silencing efficiency is around 45% for fragments ranging from 21 to 39 nucleotides, 65% for a 42‐nucleotide fragment, and around 75‐90% for fragments of 45 nucleotides or larger

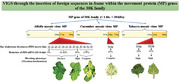

**Abbreviations**
AMV: alfalfa mosaic virus
CMV: cucumber mosaic virus
CP: coat protein
dpi: days post‐inoculation
MP: movement protein
PDS: phytoene desaturase
RDR: RNA‐dependent RNA polymerase
TMV: tobacco mosaic virus
VIGS: virus‐induced gene silencing

## 1 Introduction

An increasing number of plant viruses have been engineered to modulate gene expression for functional studies [1]. Of particular interest is their use as tools to induce endogenous gene silencing in their hosts through a strategy known as virus‐induced gene silencing (VIGS) that exploits plant defense mechanisms against RNA viruses [2]. VIGS has transformed our understanding of plant biology [3]. The VIGS method involves introducing a region of high homology to a portion of the target gene(s) into the viral genome. Upon activation of the virus‐directed posttranscriptional gene silencing or transcriptional gene silencing machinery, the host gene, part of which is integrated into the viral genome, is collaterally silenced [4, 5].

Although there are different methods to analyze gene function [6], VIGS provides a simple way to quickly silence genes and observe phenotypes. VIGS holds advantages over other gene‐silencing techniques in several contexts. These include, among others, the functional study of redundant genes with similar sequences, the study of genes that cannot be modified by gene editing techniques or those causing lethality in embryonic or juvenile stages, and the analysis of gene functionality in species lacking gene modification mechanisms [5, 7]. Furthermore, VIGS can be especially useful for exploring gene functionality in agriculturally significant species [5, 8].

To date, more than 50 VIGS vectors based on RNA and DNA viruses have been described [9]. The heterologous fragments used in each vector exhibit diverse structures and sizes, encompassing linear, sense, or antisense orientations, as well as direct or indirect repeats [10]. Typically, linear inserts range in size from 100 to 500 bp [11, 12, 13, 14], although successful silencing has been reported with inserts as small as 33 bp, which decreases the probability of silencing other host transcripts sharing unintended sequence similarity with the targeted gene(s) [15].

There are several methods for integrating the sequence of the target gene(s) into the viral genome. In general, these inserts are placed into non‐coding regions. This is the strategy employed, among others, for VIGS generated using turnip yellow mosaic virus [16] or barley stripe mosaic virus [14]. Alternatively, other approaches can be used. For instance, the whole sequence of a non‐essential viral gene can be replaced with the sequence of the target gene(s) [17]. A widespread example of such a strategy is that achieved with tobacco rattle virus [18] due to its wide host range, comparatively milder symptoms that cause in their hosts and ease of handling. The TRV system has also been used to generate robust VIGS by employing heterologous subgenomic promoters for insert expression [19]. Another less‐explored approach involves introducing the inserts into the coding sequence of a viral protein [20].

Regardless of the method, VIGS has emerged as a powerful tool for studying gene functionality in agriculturally relevant species [3]. However, generating a wide range of phenotypes with different silencing degrees can be useful for accelerating functional analysis, while studying vital genes also requires downregulation instead of completely blocking their expression. Therefore, it is crucial to develop and fine‐tune new VIGS systems to induce a particular degree of silencing of a specific gene or improve the performance of the existing viral vectors. To address this question, we focus this work on the development of new VIGS vectors by using the movement proteins (MPs) of the 30K family [21] as a platform to insert RNA sequences of genes of interest.

The 30K family of MP comprises 20 viral genera, whose MP are related to the 30 kDa MP of tobacco mosaic virus (TMV) [21, 22]. The family includes more than 500 viral species with the capacity to infect all agronomically important plant species [23, 24]. In this work, we have used alfalfa mosaic virus (AMV) as a model to develop new VIGS vectors with the potential to be applied to any virus assigned to the MP 30K family.

AMV is a member of the *Bromoviridae* family and is classified in the *Alfamovirus* genus [25]. Its genome comprises three RNA strands of positive polarity. RNA 1 and 2 are monocistronic and encode the viral replicase Subunits 1 and 2, respectively. RNA 3 is bicistronic and encodes the MP, which belongs to the 30K family, and the coat protein (CP). The latter protein is expressed from RNA 4, a subgenomic RNA derived from RNA 3 [26].

Here, we show that the AMV experimental system allows the in‐frame insertion of small sequences of interest into the coding sequence of the AMV MP without affecting protein functionality [27]. We have utilized this property to incorporate a portion of the sequence of the *Nicotiana benthamiana* phytoene desaturase (*PDS*) gene into the MP gene to induce *PDS* silencing. The AMV system was used to analyze how the insert size and the viral encapsidation process influence the silencing efficiency and how it can be used to modulate the degree of silencing. Finally, to demonstrate that this strategy could be applied to other members of the MP 30K family, the TMV and the cucumber mosaic virus (CMV) MP were modified using the same procedure as with AMV. TMV is the type species of the genus *Tobamovirus* whose members have been successfully used as VIGS vectors by utilizing the strategy of subgenomic expression, which involves duplicating the subgenomic promoter sequence [28]. This strategy is prone to undergo partial or complete loss of the inserted sequence by homologous recombination. On the other hand, CMV belongs to the genus *Cucumovirus* in the family *Bromoviridae* and has also been successfully used as a VIGS vector. Various strategies have been employed to locate inserts into RNA 2 and 3. In RNA 2, a fragment of the 2b protein sequence was replaced by the corresponding targeted gene [29]. In RNA 3, the inserts were located in the intercistronic region between the MP and CP genes [15]. In contrast, our work presents a new method that repetitively utilizes the same configuration of the MP within the 30K family to create novel VIGS vectors. Here, we demonstrated that AMV vectors with modified MP are promising VIGS tools for functional studies of host genes. By demonstrating that this strategy also works in other viruses belonging to the same 30K MP family, we extend the limited repertoire of VIGS vectors, covering a wide spectrum of hosts.

## 2 Materials and Methods

### 2.1 DNA Manipulation

#### 2.1.1 AMV Constructs for Silencing *N. tabacum PDS* Gene

The AMV cDNA corresponding to RNA 3 (cDNA3), carrying a 5′UTR wild type (GenBank: K03542.1) or the 5′UTR evolved by serial passages [27, 30], was modified to introduce the *N. tabacum PDS* (LOC107816873) sequence insert between amino acids P256 and S257 of the AMV MP. For inserts smaller than 102 bp, the following procedure was used: the AMV MP was amplified (from amino acids M1 to P256) by PCR using an antisense primer containing the corresponding *PDS* insert sequence (Table S1) and the *Nco*I and *Nhe*I restriction sites at the 5′ and 3′ ends, respectively. The digested PCR fragments were used to replace the wt MP (amino acids M1 to S256) in an AMV cDNA3 derivative containing *Nco*I and *Nhe*I sites at the start codon and after the residue S256 [31], respectively. Inserts of 102 bp or bigger were amplified by RT‐PCR using specific oligos (Table S1) carrying a *Nhe*I site at both the 5′ and 3′ termini. The *Nhe*I‐digested fragments were inserted in the AMV cDNA3 previously digested with *Nhe*I and dephosphorylated. The orientation of the inserts was verified by DNA sequencing of the clones.

#### 2.1.2 AMV Constructs for Silencing *N. benthamiana PDS* Gene

The assays in *N. benthamiana* were performed using the plasmids ptZ/cDNA1, ptZ/cDNA2, and ptZ/cDNA3, which contain AMV RNAs 1, 2, and 3, respectively [32]. The introduction of different *N. benthamiana PDS* (EU165355.1) sequences between amino acids P256 and S257 of the AMV MP was performed by PCR using specific primers that amplify the AMV cDNA3 in two fragments. The first PCR fragment contains the viral sequence from the 5′ termini to amino acid P256 of the MP. The second PCR fragment encompasses the AMV sequence from amino acid S257 of the MP to the 3′ termini. The *PDS* sequences were inserted as part of the primer sequences (see Table S1), and the assembly of two AMV cDNA3 PCR fragments plus the pTZ vector was addressed using the *Bsa*I restriction sites which generated compatible DNA ends between the two amplified fragments and with the cauliflower mosaic virus 35Sx2 promoter (5′ termini of viral sequence) and the *Solanum tuberosum* proteinase inhibitor II gene terminator (PopIt) (3′ termini of viral sequence) of pTZ plasmid, respectively. All DNA constructs were analyzed and confirmed by plasmid DNA sequencing.

#### 2.1.3 TMV and CMV Constructs for Silencing *N. benthamiana PDS* Gene

The TMV [33] and the isolate CMV‐Q infectious clones were obtained by introducing the corresponding cDNA into a modified pLX binary vector [34], which included the 35Sx2 promoter and the PopIt sequences. PCR amplification and cloning were performed using specific primers for the 5′ and 3′ ends of the viral sequences, which included nucleotide sequences compatible with the 35S and PopIt ends, respectively, engineered using the type II *Bsa*I restriction site. The *N. benthamiana PDS* sequences were inserted into the MP gene between amino acids R213 and T214 in TMV, and between H247 and E248 in CMV [35, 36]. The procedure for amplifying the TMV and CMV genomes in two fragments was carried out as described above for AMV. The first fragment for TMV contained the 5′ termini of the virus up to amino acid R213 of the MP, while the second fragment encompassed the rest of the viral genome. Similarly, for CMV, the first fragment contained the 5′ termini of the virus up to amino acid H247 of the MP, while the second fragment encompassed the rest of the viral genome. The *PDS* sequences were introduced as part of the primer sequences (Table S1), along with a *Bsa*I restriction site to fuse the two PCR fragments. All DNA constructs were analyzed and confirmed by plasmid DNA sequencing.

### 2.2 Plant Material and Growth Conditions

*Nicotiana tabacum* P12 and *N. benthamiana* plants were used*. N. tabacum* P12 are transgenic plants that constitutively express the P1 and P2 subunits of the AMV replicase [37]. Inoculation of *N. tabacum* P12 plants with AMV RNA3 is sufficient to initiate infection. *N. tabacum* plants were grown under long‐day photoperiods of 16 h light at 24°C and 8 h dark at 21°C.

Two versions of *N. benthamiana* were employed: the wild‐type version, Nb(wt), and the rdr6i‐Nb line, in which the RNA‐dependent RNA polymerase 6 (RDR6) is constitutively silenced [38]. Both *N. benthamiana* versions were grown under long‐day photoperiods, comprising 16 h of light at 25°C and 8 h of darkness at 22°C.

### 2.3 Inoculation of *N. tabacum* P12 Plants

*N. tabacum* P12 plants were inoculated with T7 RNA polymerase‐derived transcripts from various AMV RNA 3 constructs. The transcription reactions were conducted in a final volume of 20 µL, including 2 µL of 10× transcription buffer (Roche Diagnostics GmbH, Mannheim, Germany), 2 µL of 10 mM nucleoside triphosphates (NTP), 0.3 µL of Ribolock RNase A inhibitor (40 U/µL) (Thermo Fisher Scientific, Carlsbad, CA, USA), 0.4 µL of T7 RNA polymerase (50 U/µL) (TaKaRa Bio Inc., Sigha, Japan), and 250 ng of the PCR‐amplified DNA template. The PCR products utilized in the transcription reaction were generated using the sense primer representing the 5′‐terminal 17 nucleotides of AMV RNA 3 preceded by the T7 promoter sequence (Table S1). Inoculation of transcripts was carried out using carborundum. Two leaves per plant at the six‐leaf stage were inoculated by gently rubbing 10 µL of the transcription reaction mixture per leaf.

### 2.4 Inoculation of *Nicotiana benthamiana* Plants

Unlike *N. tabacum* P12 plants, the inoculation of *N. bethamiana* plants required the presence of all three viral RNAs in the inoculum. To increase the efficiency of the inoculum, the viral infection was initiated by agroinfiltration. To achieve that, *Agrobacterium tumefaciens* (strain C58), transformed with the corresponding binary plasmid, was cultured overnight in liquid Luria–Bertani (LB) medium supplemented with kanamycin and rifampicin antibiotics. Afterward, the bacterial cultures were centrifuged, and the resulting pellet was resuspended in the infiltration solution (10 mM MgCl_2_, 10 mM MES adjusted to pH 5.6) to achieve the desired final OD_600_ value of 0.2. For experiments involving AMV inoculation, which require the mix of three independent bacterial cultures (one for each viral RNA 1, 2, and 3), each culture was adjusted to an OD_600_ value of 0.2. The suspensions were gently infiltrated into the abaxial side of 2‐week‐old *N. benthamiana* leaves by applying slight pressure.

### 2.5 Total RNA Extraction and Real‐Time Quantitative Reverse Transcription PCR

Total RNA was obtained from at least three plants using TRIzol Reagent (Thermo Fisher Scientific, Carlsbad, CA, USA). DNase I treatment was applied to eliminate any remaining genomic DNA. To generate first‐strand cDNA, 0.5 µg of total RNA was reverse transcribed using RevertAid H Minus Reverse Transcriptase and oligo(dT) (Thermo Fisher Scientific). Real‐time quantitative polymerase chain reaction (qPCR) was performed using the QuantStudio 3 Real‐Time PCR machine (Applied Biosystems, Waltham, MA, USA) and PyroTaq EvaGreen qPCR Supermix (Solis BioDyne, Tartu, Estonia), along with specific oligonucleotides and recommended qPCR cycles. The initial denaturation lasted for 12 min at 95°C, followed by 40 cycles of 15 s at 95°C and 60 s at 60°C. Gene‐specific oligonucleotides (Table S1) designed using Primer3web version 4.1.0 (https://bioinfo.ut.ee/primer3) were employed to amplify the cDNA sequence of the *PDS* gene of *N. benthamiana* (EU165355.1) and *N. tabacum* (LOC107816873). Oligonucleotide efficiencies were evaluated through qRT‐PCR using 10‐fold serial dilutions of the corresponding cDNA. Each biological replicate underwent three independent runs. Protein phosphatase 2A (PP2A) was used as the endogenous control in *N. benthamiana* [39] and β‐tubulin in *N. tabacum* [40].

## 3 Results

### 3.1 Introduction of *PDS* Gene Sequence Fragments into the AMV MP Coding Sequence Results in *PDS* Silencing in *N. tabacum*

**FIGURE 1:**
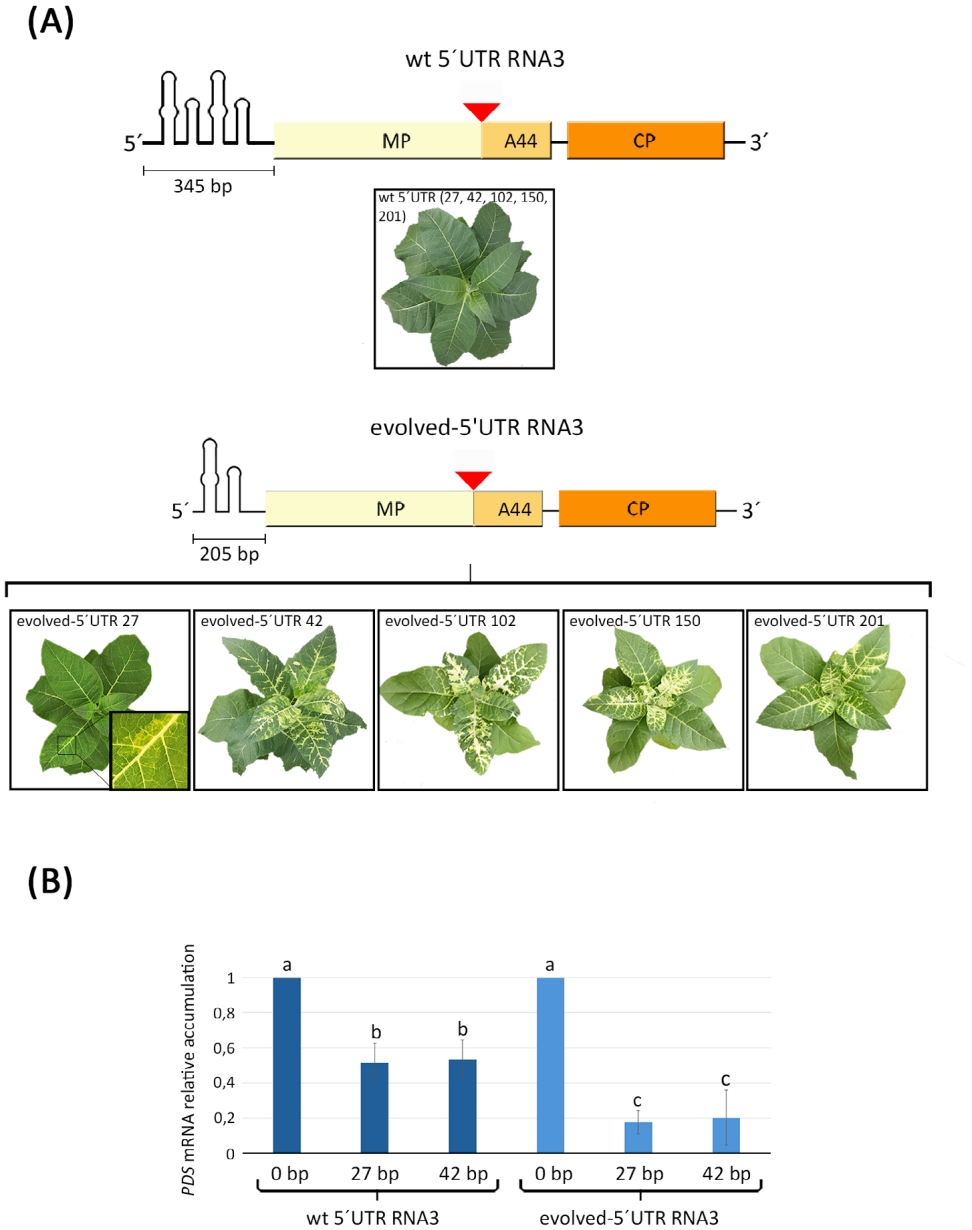
Analysis of the influence of the effect of the AMV encapsidation on the levels of *PDS* gene silencing. (A) Schematic representation of the AMV RNA 3 carrying the wild‐type (wt 5′UTR) or the evolved (evolved‐5′UTR) 5′UTR. Boxes correspond to the open reading frames of the movement protein (MP) and coat protein (CP). The red triangle indicates the point of insertion of the *PDS* fragments between the N‐terminal 256 amino acids (aa) and the C‐terminal 44 aa of the MP. The number below the 5′UTR indicates their size in nucleotides. The images below each construct display P12 plants inoculated with the AMV RNA 3 derivatives carrying 27, 42, 102, 150, or 201 bp inserts at 9 dpi. For the wt 5′UTR construct, all P12 plants showed no beaching phenotype. The name of the corresponding construct is indicated in each image. (B) The graph displays the relative accumulation of mRNA from the *N. tabacum PDS* determined by qRT‐PCR analysis. The accumulation data were determined in P12 plants either infected with the wt 5′UTR or the evolved‐5′UTR constructs without insert (0) or carrying either the 27 or the 42 bp insert, respectively at 14 dpi. Each point corresponds to three independent plants. Experiments were performed in triplicate. Statistical analysis (Student's *t* test, *p* < 0.05) of the data enabled the identification of differences between groups, which are represented in the graph by the letters a, b, and c. AMV, alfalfa mosaic virus; dpi, days post‐inoculation; *PDS*, phytoene desaturase.

To assess the potential of AMV as a VIGS vector, we inserted fragments of the *N. tabacum PDS* gene into the AMV MP coding sequence by modifying the cDNA3 of AMV (Table S2). The optimal insertion site was determined using the P12 experimental system (*N. tabacum* P12 plants constitutively expressing the P1 and P2 subunits of the AMV replicase) by performing serial deletions of the C‐terminal end of the protein until loss of function was achieved. The position for inserting heterologous sequences was determined based on the last amino acid of the mutant MP version with the largest C‐terminal deletion that did not affect its functionality [31]. Therefore, inserts were introduced between amino acids P256 and S257, as the C‐terminal 44 amino acids, while not required for systemic transport, are necessary for an efficient distal transport. An engineered *Nhe*I restriction site inserted between amino acids P256 and S257 was employed for this purpose. Two different versions of the AMV RNA 3 were used: the wild‐type virus and a version with a shorter 5′UTR RNA 3, termed evolved‐5′UTR AMV (Figure 1A, graphical scheme) obtained after serial passages in *N. tabacum*. Previous results have shown that the evolved‐5′UTR AMV version has a significantly lower encapsidation capacity [30]. This last construct was chosen to explore a potential correlation between lower encapsidation capacity and silencing activity (see Section 3.2 and Section 4). Preliminary experiments have demonstrated that the AMV MP can tolerate insertions of up to 300 bp at the above‐mentioned position without affecting viral movement or compromising the systemic spread of infection (data not shown). Based on these results, fragments of 27, 42, 102, 150, and 201 bp of the *N. tabacum PDS* gene (Table S2) were inserted in frame into the MP coding sequence of both cDNA3 versions. Therefore, the protein open reading frame remained unaltered. The P12 experimental system was used to assess the AMV constructs. AMV RNA 3 transcripts, including those with and without the inserts, were inoculated onto the leaves of 3‐week‐old *N. tabacum* P12 by gently rubbing with carborundum. The *PDS* gene encodes the PDS enzyme involved in the carotenoid biosynthesis pathway, and it is often used as a common marker for gene silencing in many plants due to the ease of detecting its visible white phenotype [28]. Starting from 9 days post‐inoculation (dpi), only the plants inoculated with the 5′‐evolved AMV version showed visible bleaching regardless of the insert size used (Table S2). This phenomenon is illustrated in Figure 1A, exemplified by the AMV variant carrying different inserts. These findings suggested that the ability of AMV to induce target gene silencing may be affected by its encapsidation capacity. To assess this hypothesis, the *PDS* mRNA levels were quantified in P12 plants inoculated with both versions of AMV RNA 3 carrying identical sequence fragments of 27 and 42 bp from the *N. tabacum PDS* gene (Figure 1B). Representative samples were collected from all plants at 14 dpi. For plants inoculated with the RNA 3 version of wild‐type AMV constructs carrying the 27 and 42 bp *PDS* inserts, *PDS* mRNA decreased by 49% and 47%, respectively, compared to the non‐insert version (Figure 1B, left). Despite having identical *PDS* insert sequences, plants inoculated with the 5′‐evolved version of RNA 3 holding an insert exhibited an 83% and 80% decrease in target mRNA, respectively, compared to the corresponding construct without any *PDS* insert (Figure 1B, right). Remarkably, the version with a reduced virus encapsidation capacity induced a higher level of target gene silencing. This data also suggests that although the wild‐type AMV version can cause some decrease in *PDS* mRNA accumulation, a higher level of silencing is required to manifest the bleaching phenotype in *N. tabacum* plants. Therefore, depending on the desired outcome, both viral vectors can be useful for gene functional studies.

### 3.2 Significant Silencing of the *N. benthamiana PDS* Was Achieved With Inserts of 21 Bp or Larger

**FIGURE 2:**
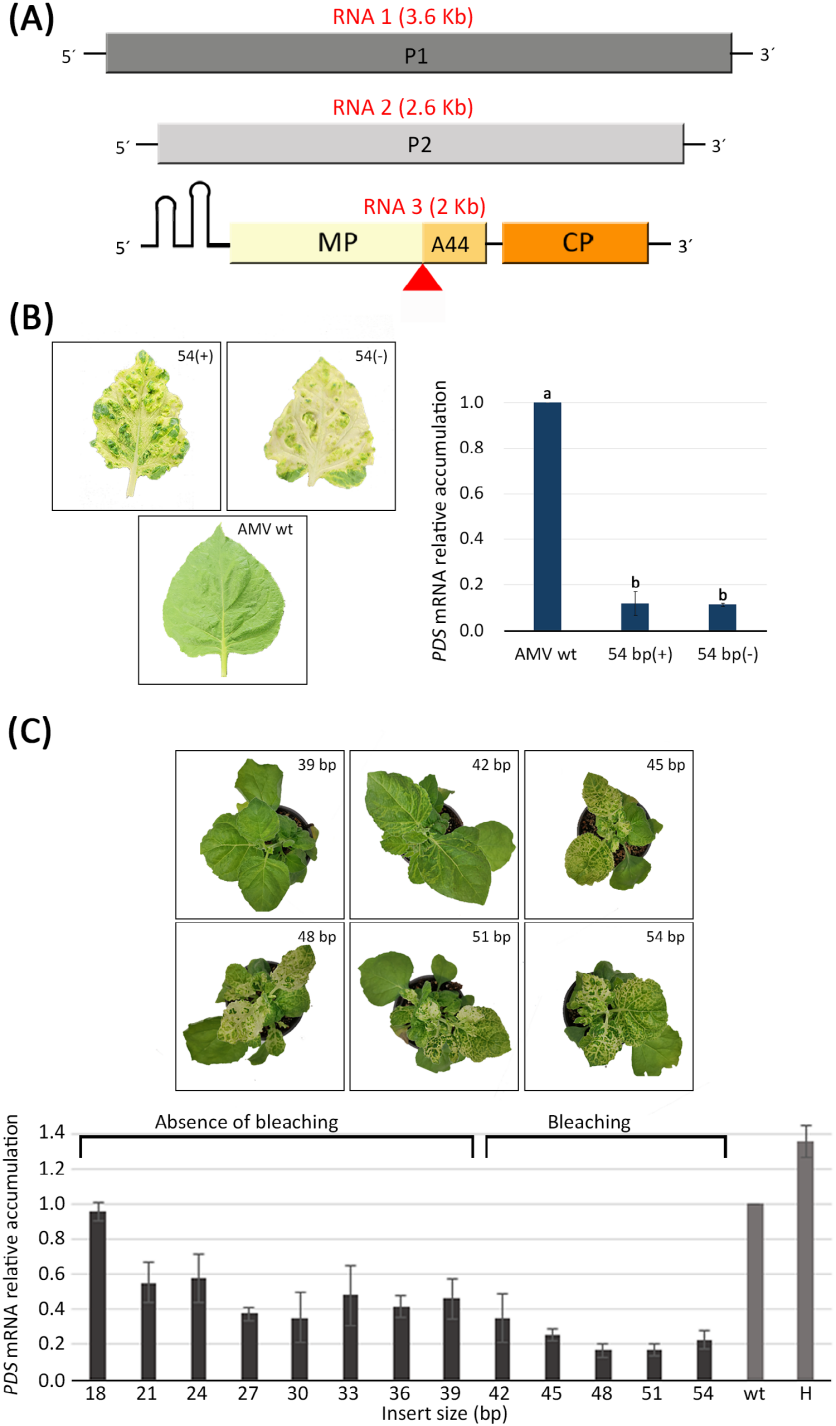
Influence of the insert size in the plant phenotype. (A) Schematic representation of the AMV genomic organization. RNA1, RNA2, and RNA3 of the virus are shown in boxes representing the open reading frames of the replicase Subunits 1 (P1) and 2 (P2), the movement protein (MP), and the coat protein (CP). The insertion site of the foreign sequences in the MP gene, between the N‐terminal 256 aa and the C‐terminal 44 aa, is indicated by a red arrowhead. Numbers in parentheses indicate the size in kb of the corresponding RNA. (B) Representative images of leaves of plants infected with AMV carrying the 54 bp inserts of positive polarity (54[+]) and negative polarity (54[−]) compared to the wild‐type virus version (AMV wt). qRT‐PCR analysis of *PDS* mRNA accumulation in AMV‐infected plants carrying the 54 bp inserts of positive polarity (54[+]) and negative polarity (54[−]) compared to the wild‐type virus version (AMV wt). (C) Representative images of AMV‐infected *N. benthamiana* plants showing no bleaching (inserts of 18–39 bp), slight bleaching (42 bp) or a white bleaching phenotype (45–54 bp). Quantification of *PDS* mRNA in AMV infected plants. qRT‐PCR analysis of the accumulation of *N. benthamiana PDS* mRNA at 10 dpi in plants inoculated with AMV constructs (three plants per construct) carrying the different *PDS* insert sizes (ranging from 18 to 54 bp). The accumulation data are normalized to those obtained with plants infected with the AMV version without an insert (wt). H indicates buffer‐inoculated plants. In the graph, the infected plants exhibiting or not bleaching phenotype are indicated. Experiments were performed in triplicate. Statistical analysis (Student's *t* test, *p* < 0.05) of the data enabled the identification of differences between groups, which are represented in the graph by the letters a and b. AMV, alfalfa mosaic virus; dpi, days post‐inoculation; *PDS*, phytoene desaturase.

In order to assess the suitability of AMV as a VIGS vector beyond the P12 experimental system, we evaluated its effectiveness in *N. benthamiana* plants as an alternative host to *N. tabacum* P12. Given the results obtained in the previous section, we used the AMV infectious clone containing an RNA 3 with the evolved‐5'UTR for the *N. benthamiana* assays (Figure 2A) [32]. The corresponding AMV cDNA3 was modified using the same procedure as above to incorporate, in this occasion, a 54 bp fragment of the *N. benthamiana PDS* gene sequence in both polarities, positive and negative. The AMV cDNA1, cDNA2, and one of the two modified cDNA3 versions with the *PDS* insert were agroinfiltrated into 3‐week‐old *N. benthamiana* plants (Figure 2A). As for the *N. tabacum* P12 plants, a bleaching phenotype was observed from 9 dpi onwards using both constructs (Figure 2B left). The accumulation of *PDS* mRNA was quantified using qRT‐PCR at 10 dpi. Plants infected with the construct carrying the 54 bp insert of the *PDS* sequence in both polarities showed an 80%–89% decrease in mRNA accumulation compared to those infected with the wild‐type virus (Figure 2B right).

**FIGURE 3:**
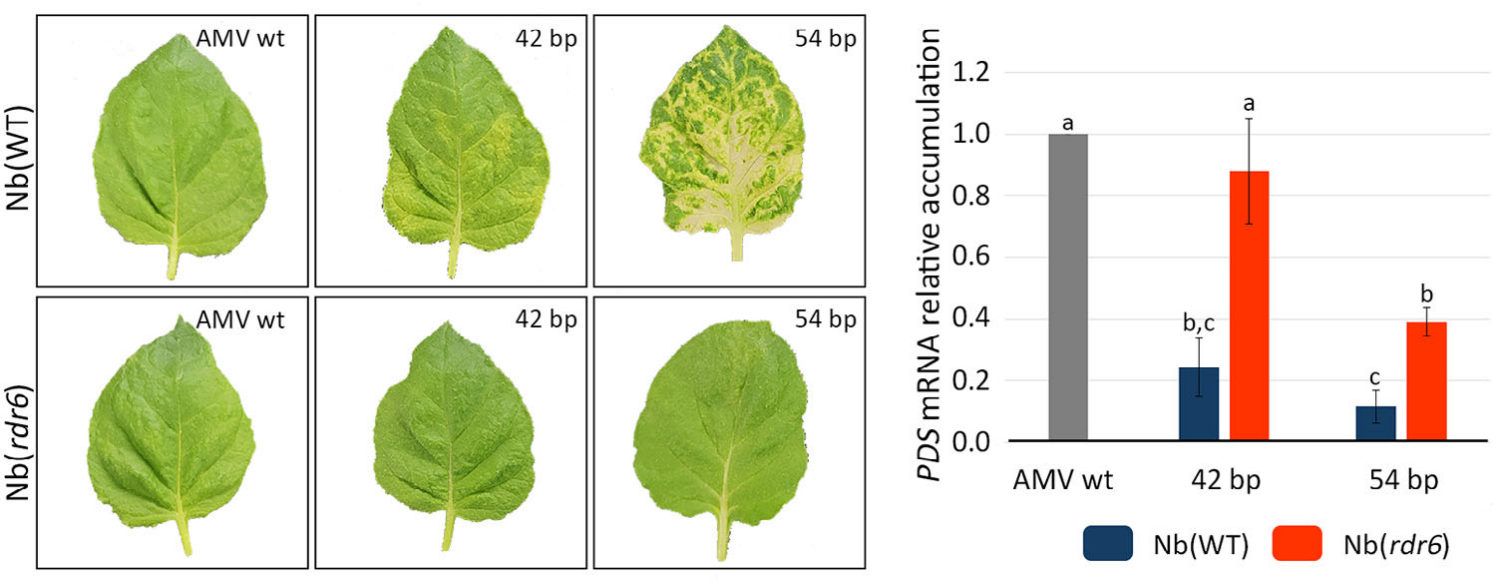
Images show the leaves of plants corresponding to *rdr*6i‐Nb line of *N. benthamiana* plants (Nb[rdr6]) and *N. benthamiana* wild type plants (Nb[wt]) infected with AMV carrying the 42 bp (42) and 54 bp (54) inserts, and the wild‐type virus version (AMV wt). Analysis of *PDS* mRNA accumulation obtained by qRT‐PCR in *rdr*6i‐Nb line of *N. benthamiana* plants (Nb[rdr6]) and *N. benthamiana* wild type plants (Nb[wt]) infected with AMV carrying the 42 bp (42) and 54 bp (54) inserts, and the wild‐type virus version (AMV wt). Experiments were performed in triplicate. Statistical analysis (Student's *t* test, *p* < 0.05) of the data enabled the identification of differences between groups, which are represented in the graph by the letters a, b, and c. AMV, alfalfa mosaic virus; *PDS*, phytoene desaturase.

To determine the insert size limit that would cause silencing and, at the same time, to correlate insert size and *PDS* gene silencing, we tested inserts of different lengths (ranging from 18 to 51 bp) and compared to the highly silencing 54 bp insert (Table 1). Plants inoculated with cDNA3 versions carrying inserts of 42 bp or larger (42, 45, 48, 51, and 54 bp) showed bleaching phenotype, while those carrying smaller inserts (18, 21, 24, 27, 30, 33, 36, and 39 bp) did not exhibit any bleaching (Table 1 and Figure 2C). At 10 dpi, systemic tissue was collected to measure the accumulation of *PDS* mRNA by qRT‐PCR. The results showed significant silencing with all constructs except for the one containing the 18 bp insert (Figure 2C). The insertion of only 21 bp of the *PDS* sequence into the AMV MP sequence was enough to cause a 45% decrease in mRNA levels. The insertion of 24, 27, 30, 33, 36, 39, 42, 45, 48, and 51 bp of the *PDS* mRNA caused a percentage drop in mRNA levels of 42, 62, 64, 52, 58, 54, 65, 75, 83, and 83, respectively (Table 1). Notably, a 65% reduction in *PDS* mRNA accumulation caused a slight onset of bleaching, characterized mainly by leaf yellowing, while a decrease of 75% or more led to a complete bleaching phenotype (Figure 2C). Interestingly, the bleaching phenotype (absence, slight, or complete bleaching) persisted in all constructs beyond 30 dpi, indicating stable *PDS* mRNA accumulation for all analyzed constructs (data not shown). The results obtained with the different *PDS* fragments indicate three different scenarios: (i) AMV constructs with *PDS* fragments up to 39 bp trigger a stable reduction of the *PDS* gene of around 50% without bleaching phenotype; (ii) AMV constructs with a *PDS* insert of 42 bp generates a silencing of around 65% and slight bleaching; and (iii) AMV construct with *PDS* fragments longer than 42 bp induced a clear bleaching phenotype, with silencing percentages of 75% or higher. To assess if these differences could be due to the amplification of the silencing response mediated by the RDR and the production of secondary siRNAs, transgenic *N. benthamiana* plants (*rdr6i*‐Nb line), in which the *RDR6* is constitutively silenced [38], were inoculated with AMV constructs carrying 42 and 54 bp of the *PDS* gene. At 10 dpi, no bleaching phenotype was observed (Figure 3 left). The qRT‐PCR quantification of the *PDS* messenger revealed a reduction of 61% for the 54 bp insert. In contrast, no significant decrease in mRNA was detected with the 42 bp insert compared to the wt version of the virus. The degree of silencing achieved with the same inserts was approximately three‐fold lower in the *rdr6i*‐Nb line than in wild‐type plants (Figure 3 right).

**TABLE 1:** Fragments of the *N. benthamiana* phytoene desaturase (*PDS*) gene inserted into the AMV, CMV, or TMV MP gene and its effects in the PDS mRNA silencing at 9 dpi.

Insert size (bp)	*PDS N. benthamiana* Insert sequence (5′‐3′)	*PDS* mRNA relative accumulation	Standard deviation	Bleaching phenotype^a^
18 (AMV)	AAGATCGAGCTGAATGAG	0.96	0.05	−
21 (AMV)	AAGATCGAGCTGAATGAGGAT	0.55	0.11	−
24 (AMV)	AAGATCGAGCTGAATGAGGATGGA	0.58	0.14	−
27 (AMV)	AAGATCGAGCTGAATGAGGATGGAAGT	0.38	0.04	−
30 (AMV)	AAAAAGATCGAGCTGAATGAGGATGGAAGT	0.36	0.14	−
33 (AMV)	ATAAAAAAGATCGAGCTGAATGAGGATGGAAGT	0.48	0.17	−
36 (AMV)	CGAATAAAAAAGATCGAGCTGAATGAGGATGGAAGT	0.42	0.06	−
39 (AMV)	TCACGAATAAAAAAGATCGAGCTGAATGAGGATGGAAGT	0.46	0.11	−
42 (AMV)	AACTCACGAATAAAAAAGATCGAGCTGAATGAGGATGGAAGT	0.35	0.14	±
45 (AMV)	CTAAACTCACGAATAAAAAAGATCGAGCTGAATGAGGATGGAAGT	0.26	0.04	+
48 (AMV)	AGACTAAACTCACGAATAAAAAAGATCGAGCTGAATGAGGATGGAAGT	0.17	0.03	+
51 (AMV)	GTCAGACTAAACTCACGAATAAAAAAGATCGAGCTGAATGAGGATGGAAGT	0.17	0.03	+
54 (AMV)	CAAGTCAGACTAAACTCACGAATAAAAAAGATCGAGCTGAATGAGGATGGAAGT	0.23 (Exp1) 0.12 (Exp2)	0.06 0.06	+
54 (−) (AMV)	ACTTCCATCCTCATTCAGCTCGATCTTTTTTATTCGTGAGTTTAGTCTGACTTG	0.11	0.01	+
54 (CMV)	GGTGGCCAAGTCAGACTAAACTCACGAATAAAAAAGATCGAGCTGAATGAGGAT	0.13	0.00	+
54 (TMV)	GGTGGCCAAGTCAGACTAAACTCACGAATAAAAAAGATCGAGCTGAATGAGGAT	0.61	0.17	−
102 (TMV)	CCTCCTGAGAGACTTTGCATGCCGATTGTGGAACATATTGAGTCAAAAGGTGGCCAAGTCAGACTAAACTCACGAATAAAAAAGATCGAGCTGAATGAGGAT	0.59	0.04	+

Abbreviations: AMV, alfalfa mosaic virus; CMV, cucumber mosaic virus; TMV, tobacco mosaic virus.

^a^ Positive (+) or negative (−) bleaching phenotype observed at 9 dpi.

Taken together, the results suggest that the size of the insert may affect the level of silencing achieved, particularly when the inserts are shorter than 54 bp, and that the insert size limit to cause an efficient silencing is 21 bp. Additionally, a decrease of more than 65% in the mRNA levels of the *PDS* gene in *N. benthamiana* is necessary to manifest the bleaching phenotype during AMV infection. However, the activity of the RDR6 was required to reach gene silencing percentages higher than 65%, suggesting that primary siRNAs derived from AMV in the absence of RDR6 do not efficiently trigger an antiviral response of silencing as reported for other VIGS systems [41].

### 3.3 The Strategy Used to Induce Gene Silencing Using AMV Can Be Applied to CMV and TMV

**FIGURE 4:**
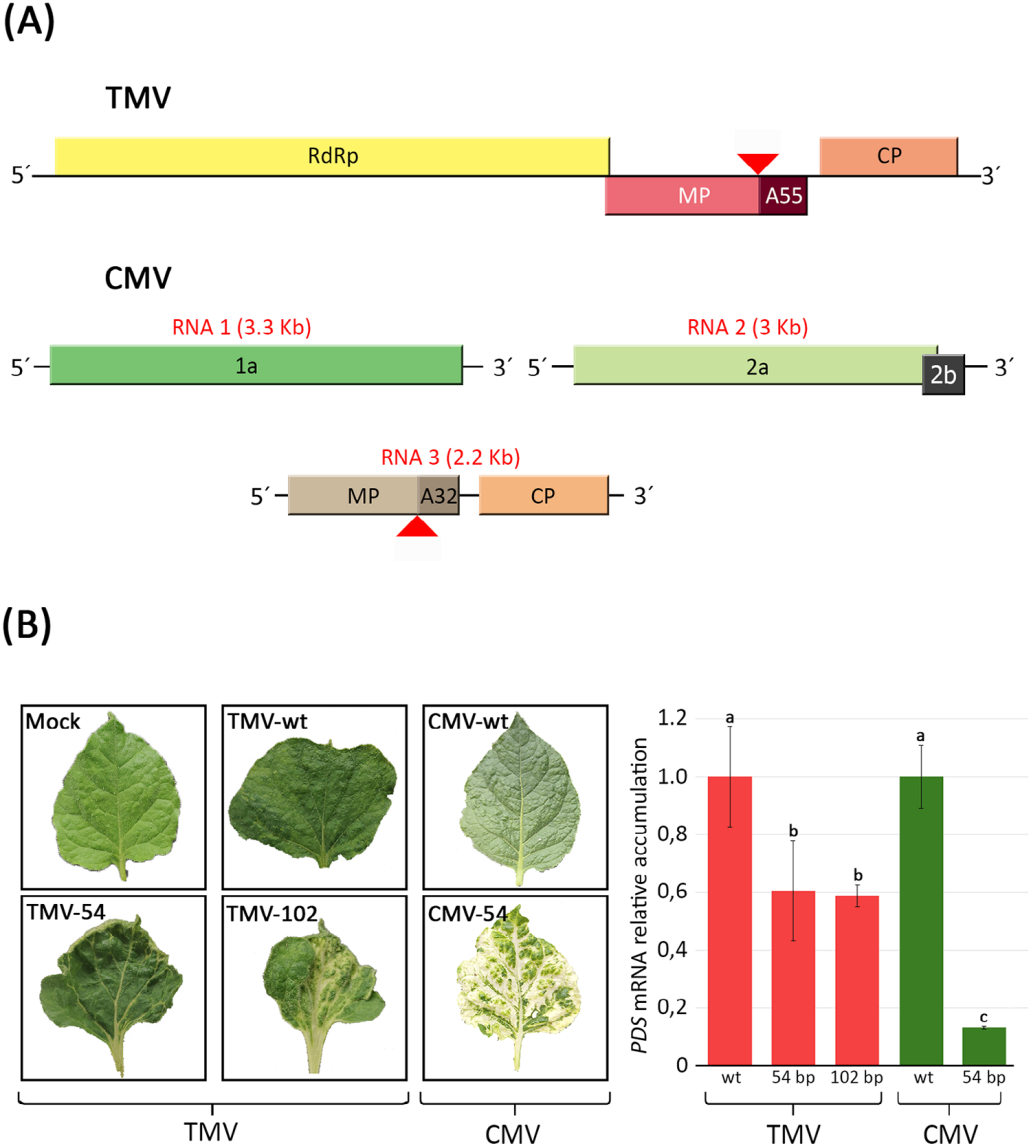
Silencing of the *N. benthamiana PDS* mRNA using TMV and CMV vectors. (A) Schematic representation of the TMV and CMV genomic organization. Boxes represent the open reading frames of the replicase (RdRp), the movement protein (MP), and the coat protein (CP) of TMV genome; and 1a, 2a, MP, and CP proteins of CMV. The insertion site of the foreign sequences in the MP genes is indicated by a red arrow. For the TMV MP, between the N‐terminal 213 aa and the C‐terminal 55 aa, and for the CMV MP, between the N‐terminal 247 aa and the C‐terminal 32 aa. (B) Images correspond to representative leaves infected with each of the viral versions. qRT‐PCR analysis of the accumulation of *N. benthamiana PDS* mRNA in plants infected with the wild‐type TMV (TMV‐wt) and with versions of the virus carrying the 54 (TMV‐54) and 102 bp (TMV‐102) *PDS* inserts; and the wild‐type CMV (CMV‐wt) and the version carrying the 54 bp insert (CMV‐54). Each construct was inoculated into three independent plants. The *PDS* mRNA accumulation is referred to the wild‐type versions of the viruses. Experiments were performed in triplicate. Statistical analysis (Student's *t* test, *p* < 0.05) of the data enabled the identification of differences between groups, which are represented in the graph by the letters a, b, and c. CMV, cucumber mosaic virus; *PDS*, phytoene desaturase; TMV, tobacco mosaic virus.

From the results above, it is evident that modifying the AMV MP to include heterologous sequences (from the target gene) is an effective strategy for inducing gene silencing. To assess the applicability of this strategy to other viruses within the MP 30K family, CMV and TMV MP were modified to include various fragments of the *N. benthamiana PDS* gene (Table 1). In the CMV MP, a 54 bp fragment of the PDS sequence was inserted between amino acids H247 and E248. In the TMV MP, either a 54 bp or an additional 102 bp insert was introduced between amino acids R213 and T214. These insertion sites were chosen based on previous findings indicating that the last 32 and 55 amino acids of the C‐terminal ends of CMV and TMV MPs, respectively, are non‐essential for both local and systemic movement [35, 36]. The reading pattern of the proteins was preserved in both cases (Figure 4A). The fragments were integrated into the MP through the amplification of the viral genome into two fragments, one of which contained the *PDS* insert followed by a *Bsa*I restriction site. The wild‐type and versions with the aforementioned inserts were agroinfiltrated into 3‐week‐old *N. benthamiana* plants. Plants inoculated with the TMV virus versions containing the *PDS* inserts showed incipient yellowing or bleaching, regardless of the insert size (Figure 4B left). However, a complete bleaching phenotype was not observed. In contrast, the CMV variant containing the *PDS* insert exhibited a clear bleaching phenotype becoming evident from 9 dpi onwards (Figure 4B left). To quantify the *PDS* silencing, the mRNA accumulation was determined via qRT‐PCR at 21 dpi for TMV and 14 dpi for CMV. The results revealed a 39% and 41% decrease in mRNA levels in plants inoculated with TMV versions carrying 54 and 102 bp inserts, respectively (Figure 4 right). Although a complete bleaching phenotype was never achieved with TMV (see Figure 4B left), the introduction of 54 and 102 bp *PDS* sequence inserts induced significant TMV‐mediated silencing. In plants infected with the CMV variant carrying the insert, the *PDS* mRNA fell 87% compared to those infected with the wild‐type version, consistent with the strong bleaching phenotype (Figure 4B). Taken together, these results indicate that the same strategy may be extended to other viruses within the 30K family, following the approach used with AMV. However, the effectiveness of the system should be evaluated by considering various factors that may require optimization.

## 4 Discussion

VIGS is particularly relevant in agricultural species for studying gene functionality [9]. This work presents a new approach for using viruses of the 30K family of MP as VIGS vectors. The MP 30K family consists of 20 viral genera and over 500 viral species capable of infecting all agronomically significant plant species [23]. In order to achieve VIGS using viruses having MPs with characteristics of the 30K family, heterologous sequences of the gene of interest were inserted into the coding sequence of these essential proteins. By using this approach, the gene silencing of the *PDS* was successfully induced in different hosts (*N. tabacum* and *N. benthamiana*). Three representative viruses of the 30K family, AMV, CMV, and TMV, were employed. The findings of this study indicate that the same approach could be applied to other members of the MP 30K family, reaching virtually all agronomically relevant hosts.

Unlike commonly used VIGS strategies, which involve the introduction of the insert(s) in non‐coding regions of the viral genome [42, 43] or replacing the sequence of a non‐essential viral gene with the sequence of the insert [44], in the present work, we have employed a novel approach based in the introduction of the insert in the coding sequence of the MP without altering its functionality. This approach will likely increase the stability of the foreign sequence, although further research is required to confirm this assumption. In addition, a previous study [31] showed that the C‐terminal 44 residues of the AMV MP are not required for the systemic transport, opening the possibility of introducing foreign sequences outside of a truncated MP gene (with a stop codon after residue 256), and thus without needing to be in‐frame with the MP gene. However, this option was discarded since, although the C‐terminal region of the AMV MP is dispensable for systemic movement, it is essential for efficient transport [31].

One of the key points of our system is the use of relatively small inserts (54 bp or less) to achieve significant silencing. However, it should be noted that the AMV system allows for the insertion of up to 201 bp. Linear inserts introduced into VIGS systems are typically between 100 and 500 bp in size [12, 45]. Nevertheless, smaller inserts have been demonstrated to induce significant silencing [15]. The present work analyzed the capacity of small inserts (between 18 and 54 bp) to induce silencing of the *N. benthamiana PDS* using AMV as a VIGS vector. Previous research has established that the minimum insert size for significant silencing of the *GFP* transgene in *N. benthamiana* using PVX as a vector is 23 bp. In the case of the endogenous *PDS* gene, the minimum insert size for silencing was determined to be 33 bp [46]. The results presented here demonstrate that an insert size of only 21 bp was sufficient to induce significant silencing (45%) of the *N. benthamiana PDS*, although it did not result in a bleaching phenotype. Insert sizes ranging from 21 to 39 bp resulted in similar *PDS* mRNA knockdown without any observable phenotypic effects. In plants, RNAi‐based tools have been extensively used in gene function studies, and some transgene‐based efficient methods for finely adjusting the degree of induced silencing have been reported [47]. It is worth noting that although a partial reduction in mRNA accumulation that occurs with small inserts in our system may not be sufficient for some studies, it may be particularly useful for some purposes, such as analyzing the functionality of lethal genes or to modulate the expression of a specific gene, resulting in different phenotypes without using time‐consuming transformation techniques.

The silencing increased to approximately 65% with a 42 bp insert, resulting in a slight bleaching phenotype, and reached levels of 75%–90%, with inserts ranging from 45 to 54 bp, causing clear bleaching effects. This suggests that a minimal silencing level is necessary to produce a bleaching phenotype using AMV system, but also that the insert size modulates the percentage of silencing. However, no direct relationship has been established between VIGS‐induced *PDS* silencing levels and the occurrence of bleaching, as the same level of mRNA accumulation can result in a different bleaching phenotype [18]. Similarly, no relationship has been found between insert size and the degree of silencing when large fragments (over 100 bp) were used [14, 48]. Therefore, the use of small inserts (45–54 bp) may help avoid unintended silencing of gene isoforms or variants. Small inserts minimize homology with nontarget genes, potentially increasing the precision in targeting specific genes or variants and reducing the risk of silencing off‐target sequences. Nonetheless, the results presented in this work suggest a relationship between size and level of silencing when the inserts are small (less than 42 bp). It is important to note that other factors, such as insert sequence or virus and host characteristics, should also be taken into consideration.

The silencing percentage obtained with the different insert sizes also points to the idea of two levels of silencing, where fragments below 42 bp induce partial silencing (around 45%), while fragments longer than 42 bp induce full silencing (about 90%). The open question is how the silencing machinery triggers this scenario and why. To answer this question, we have analyzed how RDR6, a host factor involved in the generation of secondary siRNAs and the amplification of the silencing signal [49, 50], affects *PDS* silencing. The results obtained clearly indicate that RDR6 is required to increment the *PDS* silencing percentage. In its absence, the percentage obtained with 54 bp resembles that obtained by inserts shorter than 42 bp in wild‐type plants. Indeed, the RDR6/DCL4‐dependent siRNA system has been proposed to co‐evolve as a posttranscriptional silencing mechanism to limit their activity on most endogenous transcripts and RNAs [51]. In this sense, it has been observed that multiple targeting events of miRNAs are required to silence an endogenous gene, which explains why most single‐site miRNA target transcripts, which are the vast majority of plant miRNA targets, do not spawn siRNAs [52, 53, 54]. Our results point to the same conclusion, where efficient silencing of endogenous genes requires inserts larger than 42 bp, in which RDR6 may act as a safeguard against nonspecific siRNA amplification on nontarget mRNA [54]. Interestingly, the limitation of the 42 bp insert to induce an efficient silencing of the *PDS* genes correlates with the requirement of two target sites in a single‐stranded RNA for the Argonaute ribonucleoprotein for an efficient secondary siRNAs production, as observed by Axtell et al. in 2006 [52].

Regardless of the length of infection times, the maximum level of silencing achieved with AMV, TMV, and CMV differed, even though they shared the same 54 bp insert sequence. In contrast to the CMV‐54 and AMV‐54 variants, the TMV‐54 version exhibited a considerably smaller drop (about 40%) in the *PDS* mRNA levels of the infected plants, which remained stable with prolonged infection times. Beyond the differences in the molecular characteristics of each of these viruses, the phenotypic effects developed during their infection in *N. benthamiana* varied notably. TMV generated strong symptoms, including severe leaf curling, dwarfing, and chlorosis, while the CMV variant used in this study (CMV‐Q) was asymptomatic. AMV showed intermediate symptomatology, with less severe symptoms than TMV. It cannot be excluded that the capacity of each virus to induce specific symptomatology is related to its capacity to induce gene silencing. It is also important to consider other specific characteristics of each viral vector and the different defensive responses they might induce in the host.

To date, the relationship between VIGS and encapsidation has not yet been analyzed. Encapsidation is crucial in protecting viral RNA from degradation during replication and plays a significant role in viral transmission [55]. Thus, encapsidation might reduce the availability of viral RNA to the silencing machinery, resulting in lower levels of viral RNA silencing and, in VIGS systems, of the target mRNA. In the experiments performed in this study with AMV, the versions of the virus with lower encapsidation capacity produced significantly higher drops in the target mRNA levels. Even with the same insert sequence, the drop in the level of *PDS* mRNA was 40% higher compared to the non‐encapsidation defective versions of the virus. These results indicate that encapsidation shields the virus from the silencing machinery and affects its ability to induce the silencing of the target gene. Therefore, it is pertinent to consider encapsidation as another factor to be taken into account regarding the capacity of viral vectors to induce gene silencing.

Considering the aforementioned observations, the work presented here outlines a strategy for the potential use of viruses of the MP 30K family as viral vectors to induce gene silencing. However, other important factors, such as the expressed viral symptoms, the availability and efficacy of infectious clones, the host range, and others, should be taken into account when designing a VIGS vector. The results obtained with AMV, CMV, and TMV in *N. benthamiana* and *N. tabacum* suggest that most probably any of the viruses belonging to the MP 30K family could be used as VIGS vectors, leading to the development of a large number of VIGS constructs that could be used in plants of agronomic interest. The strategy employed locates the insert in a coding region of a protein essential for viral movement, which may enhance the stability of the insert. Furthermore, the use of relatively small inserts in our system can result in a high degree of silencing, and can be fine‐tuned to achieve an intermediate degree of silencing that allows the study of genes whose knock‐out is lethal. However, this aspect should be experimentally validated when using other viruses and/or hosts. In conclusion, this research paves the way for the development of novel viral vectors for the induction of gene silencing in a range of agriculturally relevant species.

## 5 Conclusions

The incorporation of heterologous sequences into the coding sequence of the 30K family of MPs has been demonstrated to be an effective approach in *N. benthamiana* and *N. tabacum* to induce gene silencing. This strategy opens the possibility of engineering VIGS vectors using most probably any virus from the 30K family, although other considerations such as the viral symptomatology, availability of infectious clones, and host range, must also be taken into account. Remarkably, in the AMV system, we observed that the silencing activity was inversely correlated with the capacity of the viral RNA to be encapsidated. Furthermore, the capacity to induce gene silencing was correlated with the size of the insert sequence. Fragments smaller than 21 nt did not induce gene silencing, while fragments between 21 and 39 bp generated a gene silencing of 45% of the target *PDS* gene. Finally, the silencing increased to approximately 65% with a 42 bp insert and reached levels of 75–90%, with inserts ranging from 45 to 54 bp. A reduction of more than 65% in the mRNA levels of *PDS* in *N. benthamiana* is necessary to manifest the bleaching phenotype during AMV infection, and the activity of RDR6 is essential to achieve this level of silencing.

## Author Contributions

**Jesús A. Sanchez‐Navarro**: conceived and designed experiments. **David Villar‐Álvarez, Jesús A. Sanchez‐Navarro, and José A. Navarro**: performed experiments. **David Villar‐Álvarez, José A. Navarro, Jesús A. Sanchez‐Navarro, and Vicente Pallas**: analyzed the data. **Jesús A. Sanchez‐Navarro**: project supervision. **David Villar‐Álvarez**: writing–original draft. **Jesús A. Sanchez‐Navarro, José A. Navarro, and Vicente Pallas**: writing–review and editing. **Jesús A. Sanchez‐Navarro and Vicente Pallas**: funding acquisition. All authors have read and agreed to the published version of the manuscript.

## Conflicts of Interest

The authors declare no conflicts of interest.

## Supporting information



Supporting Information

## Data Availability

The data that support the findings of this study are available from the corresponding author upon reasonable request. Additional supporting information may be found online in the Supporting Information section.
