# Supplementary material for: Engineering VIGS Vectors by Modifying Movement Proteins of the 30K Family
Source: Biotechnol J. 2024 Dec 22;19(12):e202400584. doi: 10.1002/biot.202400584 (PMC11664229; doi:10.1002/biot.202400584)
Supplement: Supplementary file 1 — Supporting Information [file BIOT-19-e202400584-s001.pdf]

Supplementary Table 1. Primers used in the study.

PRIMERS FOR *PDS* SEQUENCE INSERTION

AMV

	Cod Lab	Name	Sequence
N. tabacum	3480	As_MP_27PDS	CACA**GCTAGC**_ATAAATGTCCATATATGGTTTGACAGA_ AGGACCTTCGGGAATAAA
	3479	As_MP_30PDS	CACA**GCTAGC**_ATAAATGTCCATATATGGTTTGACAGAAAA_ AGGACCTTCGGGAATAAA
	3478	As_MP_33PDS	CACA**GCTAGC**_ATAAATGTCCATATATGGTTTGACAGAAAACTG_ AGGACCTTCGGGAATAAA
	3247	As_MP_36PDS	CACA**GCTAGC**_ATAAATGTCCATATATGGTTTGACAGAAAACTGAAG_ AGGACCTTCGGGAATAAA
	3245	As_MP_39PDS	CACA**GCTAGC**_ATAAATGTCCATATATGGTTTGACAGAAAACTGAAGAAC_ AGGACCTTCGGGAATAAA
	3243	As_MP_42PDS	CACA**GCTAGC**_ATAAATGTCCATATATGGTTTGACAGAAAACTGAAGAACACA_ AGGACCTTCGGGAATAAA
	3313	As_PDS_tabacum	CACA**GCTAGC**_ATCCTGTACAATAGCTTG_
	3317	S_102PDS_tabacum	CACA**GCTAGC**_GGGTTTTATTTAGCTGGTG_
	3316	S_150PDS_tabacum	CACA**GCTAGC**_GTGCCAGGTTGTGAACCC_
	3315	S_201PDS_tabacum	CACA**GCTAGC**_AAAATATTGAAGTATCAC_
N. benthamiana	3875	S_MP_18PDS	A**CGTCTC**_AAGATCGAGCTGAATGAG_ CTTGGCGTTCCTAATACC
	3876	S_MP_21PDS	A**CGTCTC**_AAGATCGAGCTGAATGAGGAT_ CTTGGCGTTCCTAATACC
	3877	S_MP_24PDS	A**CGTCTC**_AAGATCGAGCTGAATGAGGATGGA_ CTTGGCGTTCCTAATACC
	3878	S_MP_27PDS	A**CGTCTC**_AAGATCGAGCTGAATGAGGATGGAAGTC_ TTGGCGTTCCTAATACC
	3893	As_MP_30PDS	A**CGTCTC**_GATCTTTTT_ GCCTTCGGGAATAAAAACTG
	3894	As_MP_33PDS	A**CGTCTC**_GATCTTTTTTAT_ GCCTTCGGGAATAAAAACTG
	3895	As_MP_36PDS	A**CGTCTC**_GATCTTTTTTATTCG_ GCCTTCGGGAATAAAAACTG
	3896	As_MP_39PDS	A**CGTCTC**_GATCTTTTTTATTCGTGA_ GCCTTCGGGAATAAAAACTG
	3897	As_MP_42PDS	A**CGTCTC**_GATCTTTTTTATTCGTGAGTT_ GCCTTCGGGAATAAAAACTG
	3910	As_MP_45PDS	A**CGTCTC**_GATCTTTTTTATTCGTGAGTTTAG_ GCCTTCGGGAATAAAAACTG
	3911	As_MP_48PDS	A**CGTCTC**_GATCTTTTTTATTCGTGAGTTTAGTCT_ GCCTTCGGGAATAAAAACTG
	3912	As_MP_51PDS	A**CGTCTC**_GATCTTTTTTATTCGTGAGTTTAGTCTGAC_ GCCTTCGGGAATAAAAACTG
	3796	As_MP_54PDS	A**CGTCTC**_TATTCGTGAGTTTAGTCTGACTTGGCCACC_ GCCTTCGGGAATAAAAACTG
	3797	S_MP_54PDS	A**CGTCTC**_CGAATAAAAAAGATCGAGCTGAATGAGGAT_ CTTGGCGTTCCTAATACC

TMV

	Cod Lab	Name	Sequence
N. benthamiana	3883	S_MP_54PDS_TMV	A**GGTCTC**_TATTCGTGAGTTTAGTCTGACTTGGCCACC_ TCGAGATCGAAACTTTGCAAG
	4165	S_MP_78PDS_TMV	A**GGTCTC**TCTCGA_ATTGTGGAACATATTGAGTCAAAA_ GGTGGCCAAGTCAGACTAA
	4166	S_MP_102PDS_TMV	A**GGTCTC**TCTCGA_CCTCCTGAGAGACTTTGCATGCCG_ ATTGTGGAACATATTGAGTC

CMV

	Cod Lab	Name	Sequence
N. benthamiana	4300	S_MP_54PDS_CMV	A**CGTCTC**CGAATA_AAAAAGATCGAGCTGAATGAGGAT_ GAGTCCGAGATTTTAAAG
	4301	As_MP_54PDS_CMV	A**CGTCTC**TATTCG_TGAGTTTAGTCTGACTTGGCCACC_ GTGCCCAGACGCATTTTGATTA

PRIMERS FOR qPCR QUANTIFICATION

	Cod Lab	Name	Sequence
N. benthamiana	4036	S_qPCR_PDS_benth	GCTGGCGACTACACAAAACAG
	4037	As_qPCR_PDS_benth	ACGCTTGCTTCTGCCAACTTC
N. tabacum	3924	S_qPCR_PDS_tab	CGACTCCATGGGGCATAAGT
	3925	As_qPCR_PDS_tab	TTAGTTGGGCGTGAGGAAGT

PRIMERS FOR AMV RNA3 TRANSCRIPTION

	Cod Lab	Name	Sequence
	668	S AMV-RNA3 T7	ACGTTAAGCTTAATACGACTCACTATAGTATTAATACCATTTTC
	3304	As_AMV-RNA3	GCATCCCTTAGGGGCATTCATG

Nucleotides underlined and in italics correspond to the *PDS* gene of *Nicotiana tabacum* (LOC107816873) or *Nicotiana benthamiana* (EU165355.1).Nucleotides in bold correspond to the various restriction sites used.

Supplementary Table 2. Fragments of the *N. tabacum* phytoene desaturase (*PDS*) gene inserted into the AMV cDNA3 wild-type and the version carrying the evolved-5' UTR.

**Insert size (bp)**	**Insert sequence (5'-3')**	**Bleaching phenotype[1]**	
		evolved-5´UTR	WT-5´UTR
27	ATAAATGTCCATATATGGTTTGACAGA	+	-
42	ATAAATGTCCATATATGGTTTGACAGAAAACTGAAGAACACA	+	-
102	GGGTTTTATTTAGCTGGTGACTACACAAAACAGAAATACTTG GCTTCAATGGAAGGTGCTGTCTTATCAGGAAAGCTTTGTGCC CAAGCTATTGTACAGGAT	+	-
150	TGCCAGGTTGTGAACCCTGTCGGCCCTTGCAAAGATCTCCTA TTGAGGGGTTTTATTTAGCTGGTGACTACACAAAACAGAAAT ACTTGGCTTCAATGGAAGGTGCTGTCTTATCAGGAAAGCTTT GTGCCCAAGCTATTGTACAGGAT	+	-
201	AAAATATTGAAGTATCACATTGTCAAAACTCCAAGGTCTGTT TATAAAACTGTGCCAGGTTGTGAACCCTGTCGGCCCTTGCAA AGATCTCCTATTGAGGGGTTTTATTTAGCTGGTGACTACACA AAACAGAAATACTTGGCTTCAATGGAAGGTGCTGTCTTATCA GGAAAGCTTTGTGCCCAAGCTATTGTACAGGAT	+	-

[1], positive (+) or negative (-) bleaching phenotype observed at 9 dpi in *N. tabacum* p12 plants
